# Effect of cardiac-index guided intraoperative haemodynamic therapy on postoperative outcome in non-cardiac surgery patients – a systematic review and meta-analysis

**DOI:** 10.1186/s13741-026-00760-8

**Published:** 2026-11-07

**Authors:** Philipp Kazuo Omuro, Hanns-Christian Dinges, Tobias Kammerer

**Affiliations:** Department of Anaesthesiology and Intensive Care Medicine, University of Cologne, Faculty of Medicine and University Hospital of Cologne, Kerpener Str. 62, Cologne, 50937 Germany

**Keywords:** Fluid therapy, Perioperative care, Vasopressor, Inotropic drugs, Anaesthesia, Meta-analysis, Haemodynamic monitoring

## Abstract

**Background:**

Optimal perioperative haemodynamic management is essential to reduce postoperative complications. While multiple technologies allow for advanced monitoring, clear evidence on the effectiveness of Cardiac Index-guided goal-directed therapy (CI-GDT) compared to standard care in non-cardiac surgery is lacking. This systematic review and meta-analysis aimed to investigate the impact of CI-GDT on postoperative outcome.

**Methods:**

A systematic search using PubMed and Web of Science (2010–2025) identified randomised controlled trials (RCT) comparing CI-GDT to standard care in adult non-cardiac surgery. Study selection and data extraction were conducted according to PRISMA guidelines and registered under PROSPERO (CRD420251058399). Odds ratios (OR) with 95% confidence intervals (CI) and 95% prediction intervals (PI) were calculated using a random-effects model. Risk of bias was assessed via the RoB 2 tool. Heterogeneity was quantified with I^2^, τ^2^, and Cochran’s Q. Reporting bias was evaluated by funnel plots, Egger’s test, and trim-and-fill method. Sensitivity was assessed by leave-one-out analysis.

**Results:**

Twenty-one RCTs were included, of which 20 contributed data to the meta-analysis of postoperative complications. CI-GDT significantly reduced the incidence of postoperative complications (OR .59 [.45–.78]; PI .20–1.74; I^2^ = 62.9%). Mortality did not differ significantly between groups (OR 0.77 [.57–1.05]; I^2^ = 0%). Funnel plot and Egger’s test indicated no significant publication bias (*p*=.15). Leave-one-out analysis confirmed robustness of findings. Trim-and-fill did not suggest missing studies. Certainty of evidence was graded high.

**Discussion/conclusion:**

CI-GDT was associated with a lower incidence of patients experiencing at least one postoperative complication, whereas mortality did not differ significantly. However, substantial heterogeneity and a prediction interval crossing the null indicate that the benefit may not be consistent across clinical settings. These findings do not establish an independent advantage of CI targeting over other GDT strategies.

**Supplementary Information:**

The online version contains supplementary material available at https://doi.org/10.1186/s13741-026-00760-8.

## Introduction

### Rationale

One of the key tasks of perioperative anaesthetic management is to obtain an optimal haemodynamic situation to ensure ideal organ and tissue oxygenation. The definition of “optimal” for the haemodynamic parameters remains an important research question. Since the early 2000s multiple non- and minimal-invasive devices, like the PiCCO system, FloTrac/Acumen IQ or finger cuff-based monitoring devices, to measure perioperative haemodynamics have emerged (Kaufmann et al. 2020; Kanazawa et al. 2020; Fischer and Lorne 2021; Schneck et al. 2020). Implementation of these devices in the clinical routine also resulted in broad availability of advanced haemodynamic monitoring. However, diagnostics without adequate therapy strategies cannot impact the outcome for our perioperative patients. Goal-directed therapy (GDT) is therefore a broad umbrella term encompassing markedly different haemodynamic targets and treatment algorithms(Saugel et al. 2023). The perioperative targets differ depending on the specific device, patient’s condition, as well as the clinical situation (Kaufmann et al. 2020). Unlike dynamic variables such as stroke volume variation (SVV) or pulse pressure variation (PPV), which primarily assess fluid responsiveness, cardiac index (CI) provides an estimate of blood flow relative to body surface area and may therefore serve as an additional target of haemodynamic optimisation. Importantly, CI is rarely used as an isolated therapeutic target, as most perioperative algorithms incorporated CI alongside variables such as SV, SVV, PPV, and MAP and lead to interventions including fluid administration, vasopressors, or inotropic support. We therefore deliberately focused on randomised trials in which CI constituted an explicit treatment target within the haemodynamic algorithm. The purpose of this approach was not to establish superiority of CI over other haemodynamic targets, but to characterise the evidence for this specifically defined subgroup of GDT strategies (Pinsky 2015; Funcke et al. 2024).

### Objectives

Despite many studies with different devices, algorithms, interventions, and specific groups of patients’ broad evidence considering CI-guided GDT is lacking. A critical reflection of CI-guided goal directed therapy is missing. This systematic review and meta-analysis therefore aimed to evaluate postoperative outcomes in adult patients undergoing non-cardiac surgery managed with perioperative GDT algorithms incorporating an explicit CI target compared with usual care.

## Methods

Protocol and registration: This study’s protocol was registered at PROSPERO from the National Institute for Health and Care Research of the United Kingdom on May 22nd, 2025. It can be accessed via the protocol number CRD420251058399. The study was conducted according to the PRISMA 2020 statement. As this study is a meta-analysis based on previously published data, no new human or animal participants were involved. Therefore, ethical approval and informed consent were not required.

### Eligibility criteria

Eligible studies were randomised controlled trials enrolling adult patients (≥ 18 years) undergoing elective non-cardiac surgery. Studies were required to compare a perioperative haemodynamic treatment algorithm incorporating an explicit CI target with conventional or usual care and to report the number of participants experiencing at least one postoperative complication and/or postoperative mortality. Emergency surgery, cardiac surgery, paediatric populations, studies comparing monitoring devices without a treatment algorithm, and studies without an explicit CI treatment target were excluded. Publications in English or German were eligible.

### Information sources

PubMed/MEDLINE and the Web of Science Core Collection were selected as complementary bibliographic databases. PubMed provides extensive coverage of biomedical and clinical literature using both Medical Subject Headings and free-text indexing, whereas Web of Science offers broader multidisciplinary coverage and citation-based retrieval, including journals in anaesthesiology, perioperative medicine, and surgery. The search covered publications from January 2010 to June 2025. To identify additional eligible studies, the reference lists of all included trials and relevant systematic reviews were screened manually. Embase and CENTRAL were not searched. Although the combination of PubMed, Web of Science, and manual reference screening was considered appropriate for the focused clinical question, we acknowledge that searching additional databases could have increased the sensitivity of the literature search.

### Search

The search strategy can be found in the supplement section. Boolean operators (AND/OR/NOT) were applied to combine terms. Searches were conducted in PubMed and Web of Science. The full search strategy was adapted to each database accordingly.

### Study selection

Following duplicate removal, all records were screened by title and abstract by one reviewer. ASReview Lab, using the ELAS u4 active-learning model, was used solely to prioritise the order in which records were presented for screening. The software did not independently exclude records or make final eligibility decisions. All 1,472 records were manually assessed, and potentially eligible reports underwent full-text review. Final inclusion was determined by the reviewer according to the prespecified eligibility criteria.

### Data collection process

Study selection and data extraction were performed by one reviewer. To reduce the risk of erroneous exclusion or extraction, a second author independently verified all full-text eligibility decisions and the extracted data for the primary and secondary outcomes. Disagreements were resolved through discussion.

### Data items

The primary outcome was the number of participants experiencing at least one postoperative complication, according to the composite outcome definition and postoperative observation period used in each individual trial. As outcome definitions were heterogeneous, mortality was included in the primary composite outcome only when it formed part of the respective trial-specific definition. The components of each composite outcome and the corresponding observation periods were extracted where reported. For each intervention, we additionally extracted the predefined CI target, other haemodynamic target variables, the monitoring device, and the protocol-specified use of fluids, vasopressors, and inotropic drugs. Differences in intervention protocols were considered when interpreting clinical heterogeneity across studies. Mortality was additionally extracted and analysed separately as a secondary outcome, using the follow-up period reported by each trial.

### Study risk of bias

All included studies were assessed by one reviewer using the Cochrane Risk of Bias 2 (RoB 2) tool, supported by software-assisted guidance for domain-level judgement (Sterne et al. 2019). The following sections were analysed for each study: randomisation process, Deviations from the intended interventions, missing outcome data, measurement of the outcome and selection of the reported results. Risk of bias was categorised as low risk, some concerns and high risk.

### Effect measures

The primary effect measure was the odds ratio (OR) for experiencing at least one postoperative complication, based on the trial-specific composite outcome definitions and observation periods. Postoperative mortality was analysed separately as a secondary outcome. As the reported mortality time points varied across studies, the mortality outcome was based on the follow-up period reported in each individual trial. Pooled ORs were reported with 95% confidence intervals and 95% prediction intervals.

### Synthesis methods

Following study selection and data extraction, all relevant data were organised using Microsoft Excel for Mac (version 16.77.1). If absolute frequencies were not reported (e.g., number of patients without complications), they were calculated using corresponding reported values (e.g., number with complications or proportions). The final dataset was initially explored using IBM SPSS Statistics (Version 29.0.2.0; IBM Corp., Armonk, NY, USA). Subsequent analyses, including calculation of effect sizes and meta-analytic models, were performed in RStudio (Version 2025.05.0 + 496; Posit Software, PBC, Boston, MA, USA) running R (Version 2025.05.0 + 496; R Foundation for Statistical Computing, Vienna, Austria). Data handling and preprocessing were conducted using the dplyr and readxl packages, while meta-analytic computations and forest plot generation were performed using the meta package. Meta-analyses were conducted using a random-effects model based on inverse-variance weighting. Between-study heterogeneity was quantified using Cochran’s Q statistic, the I^2^ statistic, and the between-study variance (τ^2^). In addition to conventional heterogeneity metrics, a 95% prediction interval (PI) was calculated for each meta-analysis. Whereas the 95% confidence interval (CI) around the pooled effect reflects the precision of the estimated average effect, the PI provides an estimate of the range within which the true effect of a future study is expected to lie, considering both within-study and between-study variability. Consequently, the PI offers a more clinically meaningful measure of dispersion, particularly when heterogeneity is substantial. A wide PI that includes the null effect suggests that, although the mean pooled effect may be statistically significant, real-world effects across different clinical settings are likely to vary and may not consistently demonstrate benefit. In contrast, a narrow PI that does not cross the line of no effect indicates a robust and potentially generalisable association. A two-tailed *p*-value < 0.05 was considered statistically significant for all tests.

#### Reporting bias

Potential reporting bias was assessed visually through funnel plots and statistically via Egger’s test for funnel plot asymmetry. For each outcome, a trim-and-fill procedure was performed to estimate the potential impact of missing studies on the pooled effect estimate.

#### Certainty assessment

Certainty was analysed by one investigator and GRADEpro GDT-Tool. Study design, risk of bias, inconsistency, indirectness, missing accuracy and other factors were considered, if applicable.

#### Sensitivity analysis

To evaluate the robustness of the pooled estimates, a leave-one-out sensitivity analysis was performed. In this analysis, the meta-analysis was recalculated iteratively with one study removed at a time to assess the influence of individual studies on the overall effect size.

## Results

### Study selection

**Fig. 1:**
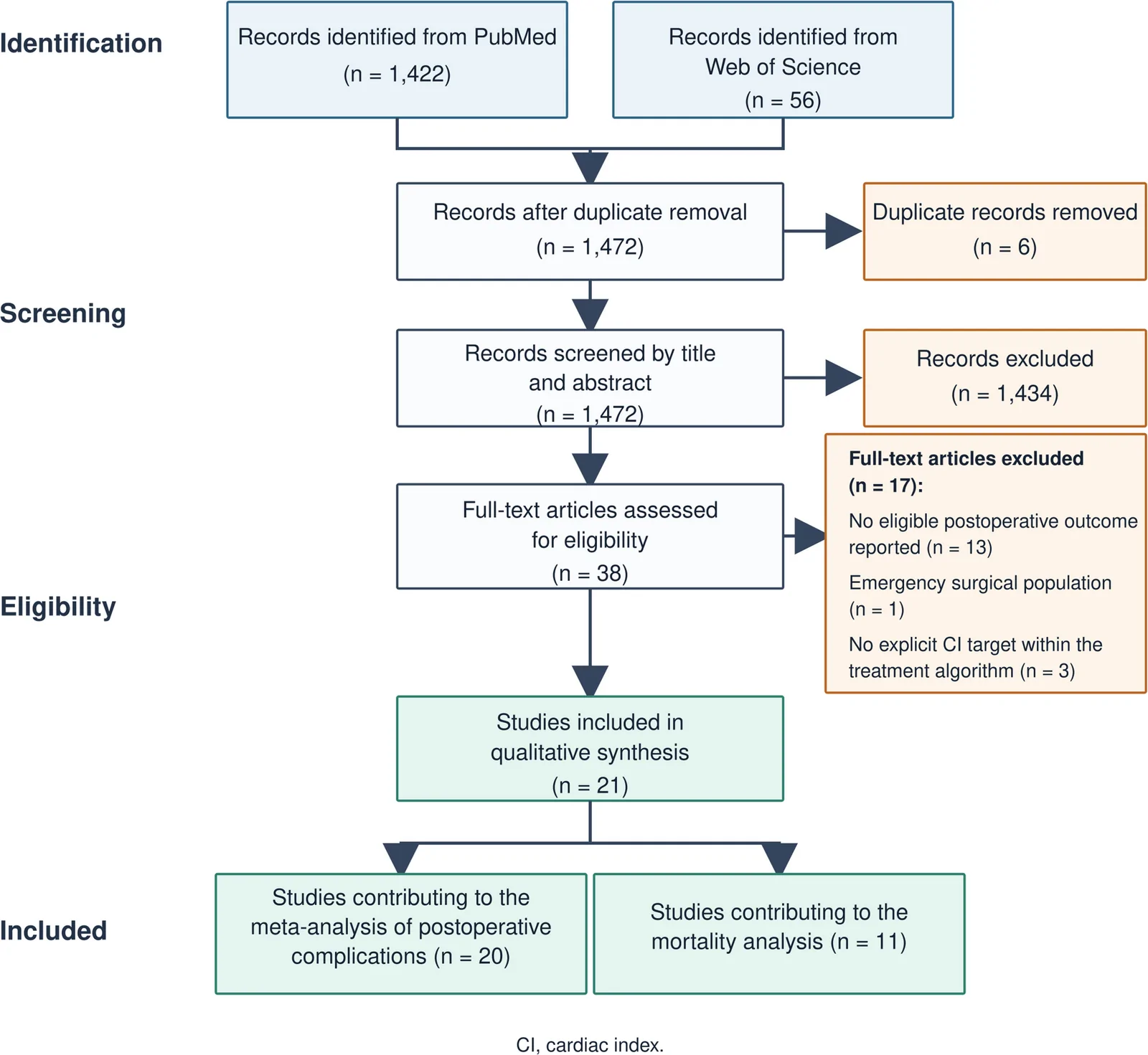
PRISMA flow diagram of the literature search and study-selection process. CI, cardiac index

The database searches identified 1,478 records. After removal of six duplicates, 1,472 records were screened by title and abstract, of which 1,434 were excluded. Thirty-eight full-text articles were assessed for eligibility. Seventeen articles were excluded because no eligible postoperative outcome was reported (*n* = 13), the study exclusively included an emergency surgical population (*n* = 1), or the intervention did not incorporate an explicit CI treatment target (*n* = 3). Finally, 21 studies were included in the qualitative synthesis. Of these, 20 contributed data to the meta-analysis of postoperative complications and 11 to the mortality analysis (Fig. 1).

### Study characteristics

The cumulative population of the 21 included studies was *N* = 3204. CI-GDT was used in *n* = 1608 cases. Cumulative control group (CON) was *n* = 1596. At least one complication was seen in *n* = 1433 cases (*n* = 672 in CI-GDT vs. *n* = 761 in CON). 12 studies were mainly conducted in abdominal and/or visceral surgery with 8 studies being labelled as “major abdominal” and 1 study reported from oesophageal surgery, 4 studies in other abdominal surgery. The remaining studies included patients undergoing neuro-, vascular, head and neck, orthopaedic, urologic, gynaecologic, as well as thoracic surgery (Table 1). Patients in the analysis by Calvo-Vecino et al. also mainly experienced abdominal surgery (~ 75%). 11 studies reported mortality as a separate endpoint. Overall *n* = 40 of *n* = 1205 patients died in the intervention and *n* = 56 of *n* = 1270 in the control group.

**Table 1:** Included study, study design, population and used monitoring devices.RCT = randomised controlled trial. y.o. = years old. GI = gastrointestinal

Study	Design	Study population	Monitoring Device
Mayer et al., (2010)	RCT	major abdominal	FloTrac/Vigileo
Van Der Linden et al., (2010)	RCT	peripheral artery	FloTrac/Vigileo
Salzwedel et al., (2013)	RCT	major abdominal	ProAQT, Pulsion
Zheng et al., (2013)	RCT	60–80 y.o., GI surgery	FloTrac/Vigileo
Pestaña et al., ( 2014)	RCT	major abdominal	﻿NICOM™
Funk et al., (2015)	RCT	mastectomy (women only)	FloTrac/Vigileo
Broch et al., (2016)	RCT	major abdominal	ccNexfin
Stens et al., (2017)	RCT	moderate risk abdominal	ccNexfin
Elgendy et al., (2017)	RCT	major abdominal	FloTrac/Vigileo
Luo et al., (2017)	RCT	high risk patients, intracranial	FloTrac/Vigileo
Kim et al., (2018)	RCT	free flap head and neck cancer	FloTrac
Calvo-Vecino et al., (2018)	RCT	low-moderate risk surgery	CardioQ*
Kaufmann et al., (2018)	RCT	cemented hip arthroplasty	Deltex Medical Doppler
Szturz et al., (2019)	RCT	Intermediat-risk GI surgery	CardioQ Esophageal Doppler
Bahlmann et al., (2019)	RCT	esophagectomy	FloTrac
Nicklas et al., (2020)	RCT	major abdominal	CNAP-System (baseline), ProAQT, Pulsion
Arslan-Carlon et al., (2020)	RCT	radical cystectomy	FloTrac
de Waal et al., (2021)	RCT	major abdominal	FloTrac/Vigileo
Li et al., (2021)	RCT	thoracoscopic lobectomy	﻿NICOM™
Hrdy et al., (2023)	RCT	neurosurgery	﻿Starling™ SV haemodynamic monitor, Cheetah
Funcke et al., (2024)	RCT	major abdominal	ProAQT, Pulsion

### Risk of bias

**Fig. 2:**
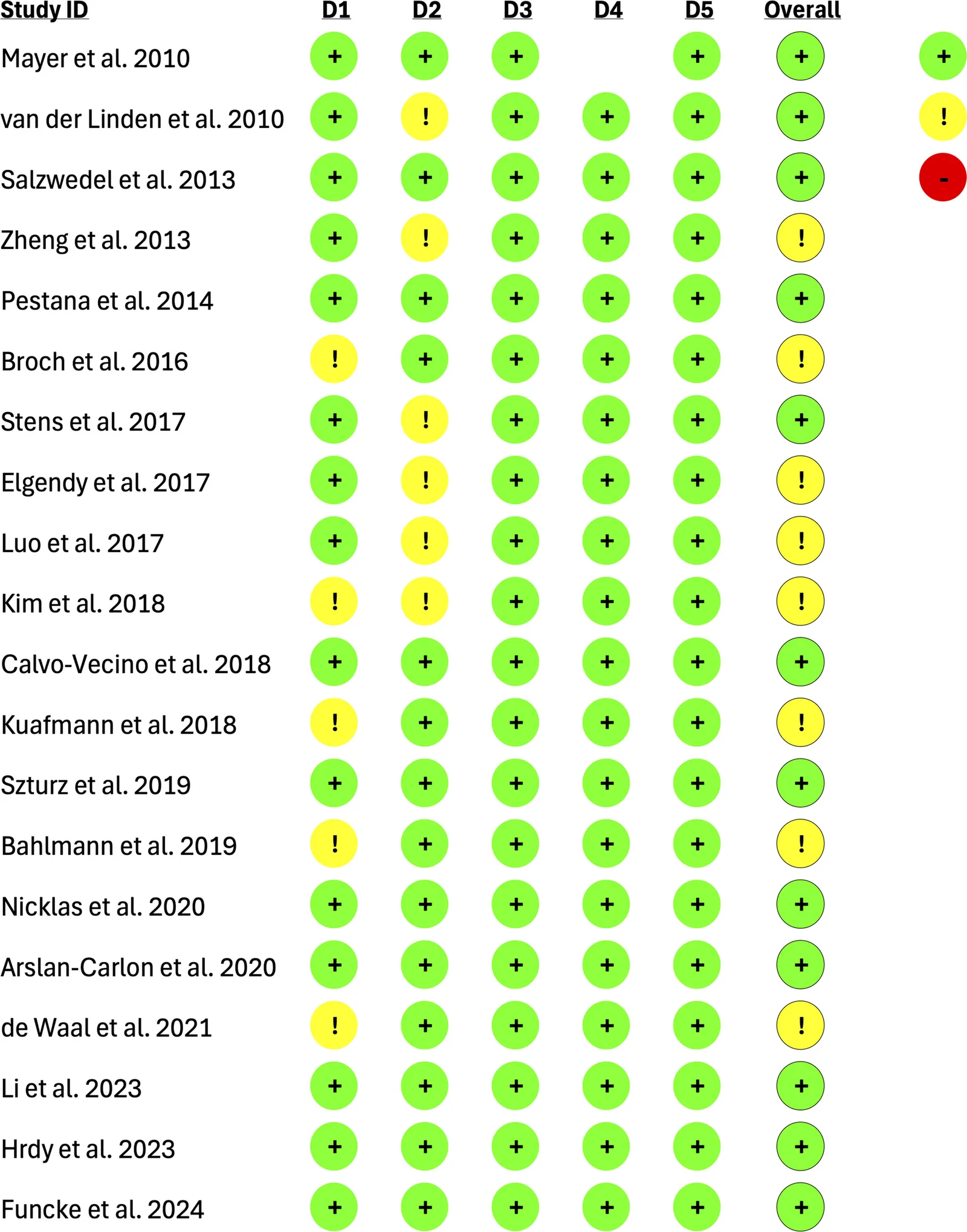
Risk of bias assessment by the Cochrane Collaboration’s RoB2-tool (Sterne et al. 2019). Risk of bias: + low risk, ! some concerns,—high risk. D1 = Randomisation process. D2 = Deviations from the intended interventions. D3 = Missing outcome data. D4 = Measurement of the outcome. D5 = Selection of the reported results

Risk of bias analysis revealed overall low risk of bias in 12 studies, as well as some concerns in 9 studies (Fig. 2).

### Results of the individual studies

#### Therapy strategies

CI targets were classified into five mutually exclusive categories: sixteen studies used a fixed threshold of 2.5 L/min/m^2^ (> or ≥ according to the individual protocol), one used ≥ 2.2 L/min/m^2^ (Funk et al., 2015), two combined an individual preload-optimised CI target with a minimum of 2.5 L/min/m^2^ (Salzwedel et al., 2013; Funcke et al., 2024), one targeted preoperative resting CI (Nicklas et al., 2020), and one used age-specific thresholds (de Waal et al., 2021; Table 2). These categories account for all 21 studies, with each study counted once. Two studies used Theodrenalin/Cafedrin, Ephedrine or Dopamine for haemodynamic optimisation. One study did not report inotropic drugs. Additionally, n = 12 studies used dynamic parameters (PPV/SVV) for guidance of therapy as seen in Table 2.

**Table 2:** List of included studies and parameters for therapy guidance and the used inotropic drugs

Study	CI target [L/min/m^2^]	other Target Variables	Inotropic drugs
Mayer et al., (2010)	> 2.5	MAP > 65 mmHg, SVI </>35 ml/m^2^	Dobutamine
Van Der Linden et al., (2010)	> 2.5	CVP < 15 mmHg	Dobutamine
Salzwedel et al., (2013)	Individ.^a^ min. 2.5	PPV < 10%, MAP > 65 mmHg	Dobutamine
Zheng et al., (2013)	≥ 2.5	SVI > 35, SVV < 12%, MAP > 65 mmHg	Dopamine
Pestaña et al., (2014)	≥ 2.5	MAP ≥ 65 mmHg	Dobutamine
Funk et al., (2015)	≥ 2.2	SVV < 13%, MAP > 60 mmHg	Ephedrine
Broch et al., (2016)	> 2.5	MAP ≥ 65 mmHg, CVP ≥ 12 mmHg, PPV < 10%	Dobutamine, Theodrenalin/Cafedrin
Stens et al., (2017)	> 2.5	PPV < 12%, MAP > 70 mmHg	Dobutamine
Elgendy et al., (2017)	> 2.5	SVV < 12%, MAP > 65 mmHg	Dobutamine
Luo et al., (2017)	> 2.5	SVV < 15%	Dobutamine
Kim et al., (2018)	≥ 2.5	SVV < 12%, MAP ≥ 65 mmHg	Dobutamine
Calvo-Vecino et al., (2018)	> 2.5	∆ SV < 10%, MAP ≥ 65 mmHg	Dobutamine
Kaufmann et al., (2018)	> 2.5	∆ SV < 10%, MAP > 70 mmHg	Dobutamine
Szturz et al., (2019)	> 2.5	SVRI 1000–1800, FTc, PV	Dobutamine
Bahlmann et al., (2019)	> 2.5	∆ SV < 10%	Dobutamine
Nicklas et al., (2020)	Preoperative resting CI^b^		Dobutamine
Arslan-Carlon et al., (2020)	> 2.5	MAP > 60 mmHg, SVV < 12%, ∆ SV < 10%	undefined
de Waal et al., (2021)	Age-specific^c^	SVV < 12%, PLR	Dobutamine
Li et al., (2021)	≥ 2.5	SVV 10–13%	Dobutamine
Hrdy et al., (2023)	> 2.5	SVV < 15%, MAP > 65 mmHg	Dobutamine
Funcke et al., (2024)	Individ.^a^ min. 2.5	PPV < 12%	Dobutamine

*CI* cardiac index, *SVI* Stroke Volume Index, *MAP* Mean Arterial Pressure, *CVP* Central Venous Pressure, *SVV* Stroke Volume Variation, *PPV* Pulse Pressure Variation, *SV* Stroke Volume, *SVRI* Systemic Vascular Resistance Index, *FTc* Flow Time corrected, *PV* Peak Velocity *Individ.* individualised

^a^Individual CI determined after preload optimisation, with a minimum of 2.5 L/min/m^2^ (Salzwedel et al., 2013; Funcke et al., 2024)

^b^Preoperative resting CI (Nicklas et al., 2020)

^c^ Age-specific CI targets: ≥ 2.8 L/min/m^2^ for age < 60 years, ≥ 2.6 L/min/m^2^ for age ≥ 60 and < 75 years, and ≥ 2.4 L/min/m^2^ for age ≥ 75 years (de Waal et al., 2021)

#### Outcome definitions for postoperative complications

As no standardised definition of postoperative complications existed composite outcome for ≥ 1 complication consisted of heterogeneous variables. Most authors reported mortality, cardiovascular, pulmonary, renal and infectious complications. The number of items considering the composition of “outcome” ranged from 4 to 39 (see supplement).

### Results of synthesis

#### Incidence of postoperative complications

**Fig. 3:**
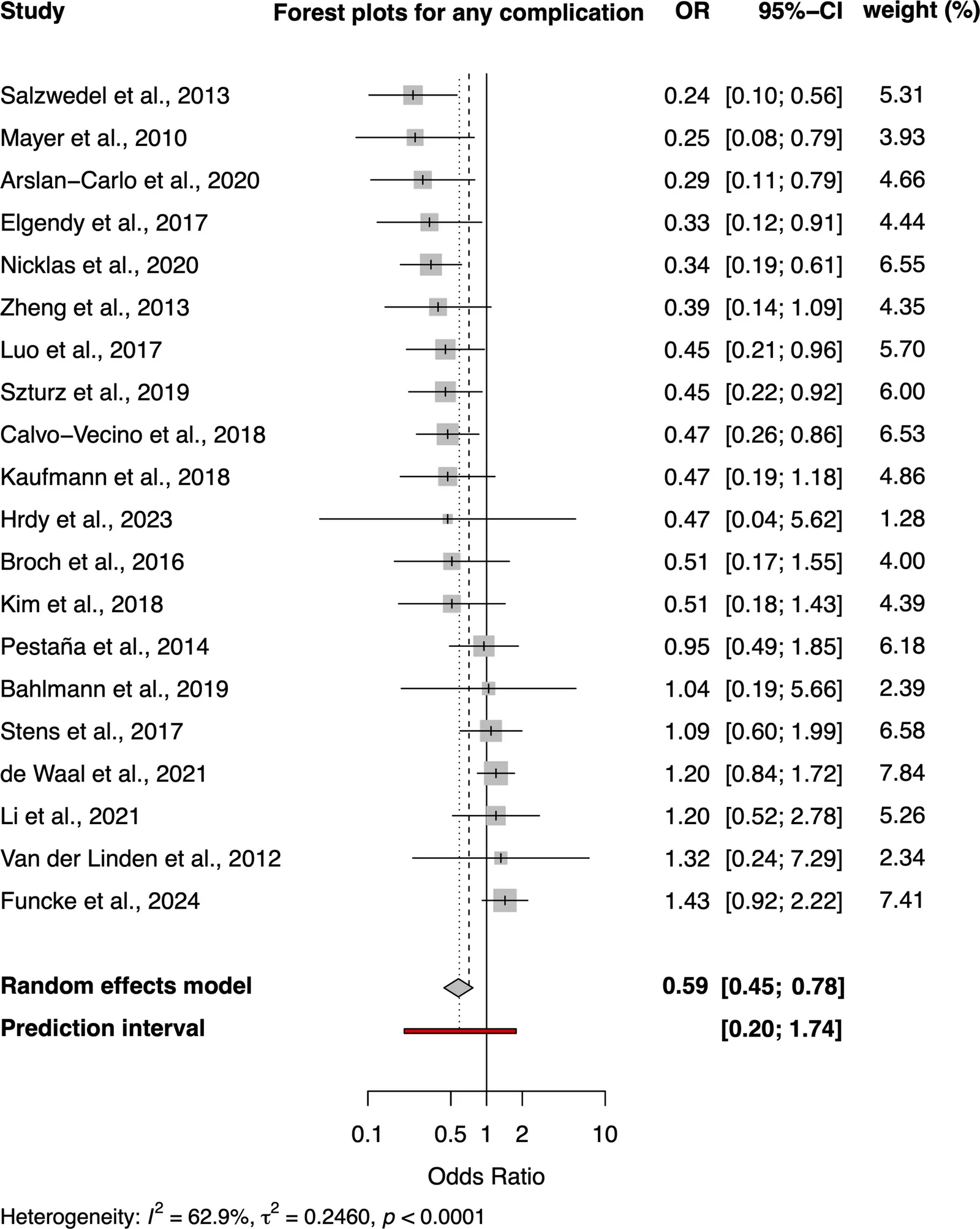
Forest Plot of the effect of Cardiac-Index guided perioperative therapy on overall complications compared to usual care. OR = Odds Ratio. 95% Confidence and Prediction Intervals

The meta-analytic model showed an OR of 0.59 [0.45-0.78] for patients experiencing at least one complication under CI-GDT compared to control (Fig. 3). Prediction interval was 0.20–1.74. Heterogeneity was substantial, with an I^2^ of 62.9% and a statistically significant Cochran’s Q test (*p* < 0.0001), indicating moderate to considerable variability in effect sizes between studies. The between-study variance (τ^2^) was estimated at 0.246. A random-effects model was therefore used for all analyses, and 95% prediction intervals were reported accordingly.

#### Mortality

**Fig. 4:**
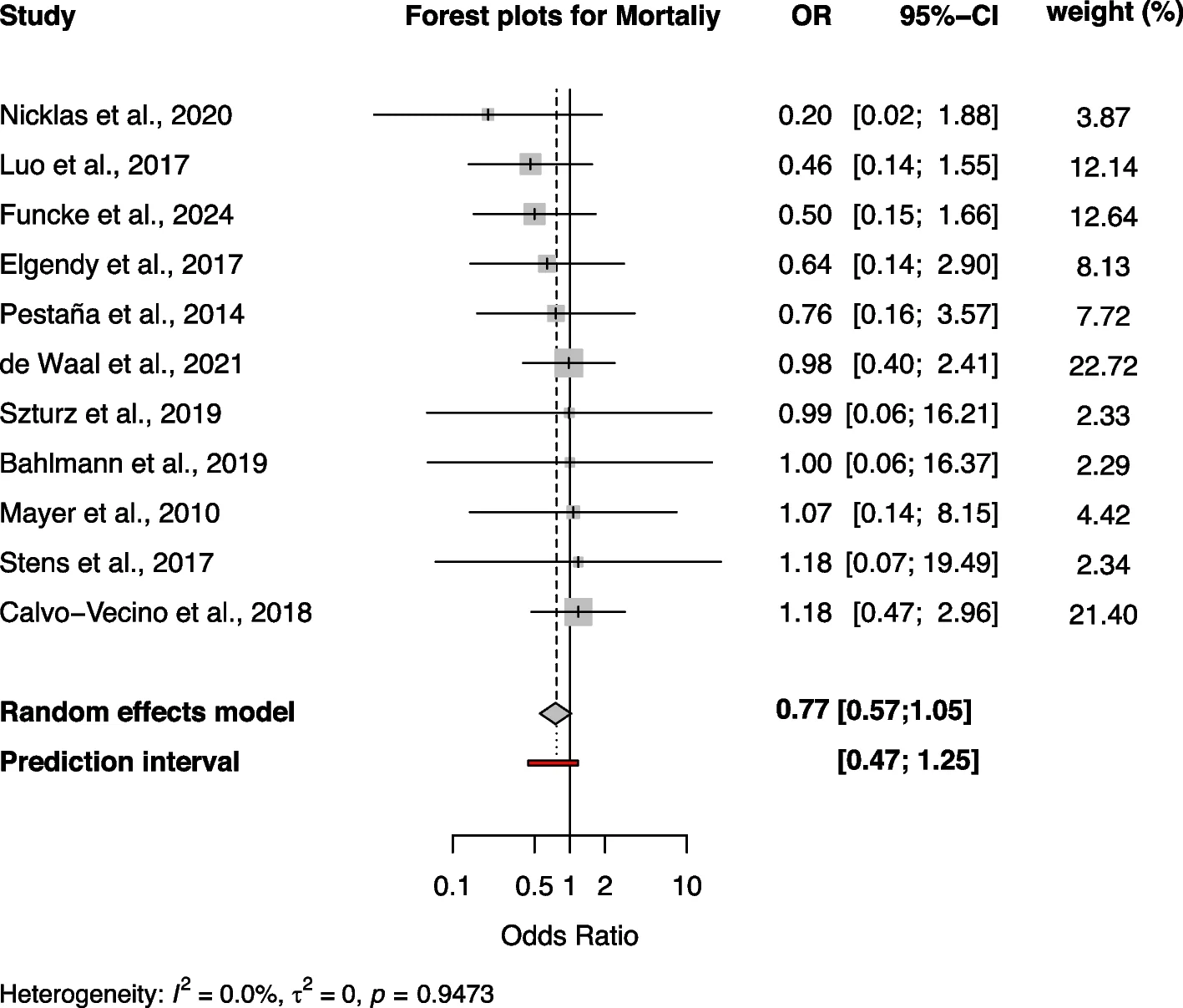
Forest Plot of Mortality of patients under Cardiac-Index guided perioperative Therapy and control. OR = Odds Ratio. 95% Confidence and Prediction Intervals

Overall mortality was comparable between patients under CI-guided perioperative therapy and patients under control OR 0.77 [0.57–1.05]; *p* > 0.05 (Fig. 4). 95% Prediction interval for mortality was 0.47–1.25. Heterogeneity for the mortality outcome was negligible (I^2^ = 0.0%, τ^2^ = 0, *p* = 0.947), indicating no observed between-study heterogeneity. Therefore, variation in the estimated effects is likely due to within-study sampling error alone.

### Reporting bias

Reporting bias was evaluated visually using a funnel plot and statistically by Egger’s test for asymmetry (see supplement). The funnel plot appeared symmetric, and Egger’s test did not indicate significant asymmetry (z = − 1.44, *p* = 0.15), suggesting no strong evidence of small-study effects.

To further explore the robustness of the meta-analytic findings, a leave-one-out sensitivity analysis was performed (see supplement). In this approach, the meta-analysis was recalculated repeatedly while omitting one study at a time. None of the exclusions substantially altered the overall effect estimate or statistical significance. The pooled log odds ratios ranged from − 0.47 to − 0.60 across all iterations, and all models remained statistically significant (p < 0.005). This indicates that the overall findings were not driven by any single study.

Additionally, a trim-and-fill analysis was conducted, which did not impute any potentially missing studies, further supporting the absence of substantial publication bias.

### Certainty of evidence

Certainty analysis revealed a high certainty of evidence regarding the endpoint any complication (*n* = 20 studies) and mortality (*n* = 11 studies) in agreement between investigator and GRADEpro GDT-tool.

## Discussion

The main finding of this meta-analysis was that GDT algorithms incorporating an explicit CI target were associated with a lower incidence of patients experiencing at least one postoperative complication, based on the composite outcome definitions and observation periods used in the individual trials. Mortality, analysed separately as a secondary outcome, did not differ significantly between the intervention and control groups. However, the heterogeneous definitions and observation periods of both postoperative complications and mortality limit the comparability of the included studies and should be considered when interpreting the pooled estimates. Considering mortality, no difference was seen between the groups. This is consistent with the results of the meta-analysis of Messina et al. (Messina et al. 2021). In their study Goal-directed therapy reduced postoperative complications without showing impact on mortality. However, Messina et al. included different therapy strategies (Messina et al. 2021). Only one included study used CI as a target parameter as the others guided therapy e.g., by dynamic parameters, SV or CVP (Messina et al. 2021). Ripollés-Melchior et al. showed in their meta-analysis no reduction of postoperative complications in patients with GDT, despite reduced mortality compared to control (Ripollés-Melchor et al. 2016). The included studies used DO (Kanazawa et al. 2020), SV and SVI as target parameters. The CI-guided study by Pestaña et al. was also included in their analysis (Pestaña et al. 2014). Li et al. meta-analysed GDT in patients undergoing thoracic surgery with OLV (one lung ventilation) (Li et al. 2023). Their results showed a superiority of GDT considering postoperative pulmonary complications (PPC) and anastomotic leakage.

Our study is the first meta-analysis specifically focusing on perioperative GDT algorithms incorporating an explicit CI target. Differences between previous meta-analyses should, however, not be attributed solely to the haemodynamic variables targeted by the respective GDT algorithms. The included patient populations, surgical procedures, baseline risk, intervention protocols, comparators, and definitions and time frames of postoperative morbidity differed substantially across reviews. Thus, the divergent pooled estimates may reflect both intervention and non-intervention heterogeneity (Messina et al. 2021; Ripollés-Melchor et al. 2016; Li et al. 2023). Consequently, the present analysis cannot determine whether targeting CI itself provides an incremental benefit over GDT strategies based on SV, SVV, PPV, or MAP. These findings emphasise the importance of precisely defining the individual targets and therapeutic components of GDT when comparing studies and translating their results into clinical practice (Saugel et al. 2023).

The definition of postoperative complications varied substantially across the included studies, thereby limiting direct comparability. Although most studies assessed cardiovascular, pulmonary, renal, and infectious complications, the individual components and definitions differed. Pulmonary complications, for example, variously included pneumonia, atelectasis, pneumothorax, or combinations thereof. Standardised outcome frameworks may improve comparability between future trials. The European Perioperative Clinical Outcome (EPCO) definitions provide standardised definitions for commonly reported perioperative outcomes and were applied in studies such as those by Nicklas et al. and Funcke et al. (Funcke et al. 2024; Nicklas et al. 2020; Jammer et al. 2015). In addition, the Core Outcome Measures for Perioperative and Anaesthetic Care (COMPAC) initiative defined a consensus-based core outcome set comprising mortality and survival, major perioperative complications or adverse events, resource use, short-term recovery, and longer-term recovery (Boney et al. 2022). Future perioperative trials should combine a consistent core outcome set, such as COMPAC, with standardised definitions and observation periods, such as those proposed by EPCO.

The literature search was restricted to PubMed and Web of Science and did not include Embase or CENTRAL. Although the two databases provide complementary coverage and reference lists of included trials and relevant systematic reviews were screened manually, eligible studies indexed exclusively in other databases may have been missed. The findings should therefore be interpreted considering potential database-selection bias.

From a clinical perspective, our findings should not be interpreted as evidence supporting routine implementation of CI-targeted therapy in all patients undergoing non-cardiac surgery. Although the pooled estimate suggested fewer patients experiencing postoperative complications, heterogeneity was substantial and the 95% prediction interval crossed the line of no effect. The observation periods for postoperative complications and mortality were not uniform across the included trials. Moreover, mortality was included in some trial-specific composite morbidity outcomes but not in others. Although mortality was additionally analysed as a separate outcome, these differences introduce clinical heterogeneity and limit direct comparability across studies.

Moreover, because CI was embedded within multimodal haemodynamic algorithms, the present analysis cannot isolate the effect of CI monitoring or determine whether adding a CI target provides benefit beyond contemporary strategies targeting SV, dynamic preload variables, and arterial pressure. This is particularly relevant in the context of the large OPTIMISE II trial, which evaluated a related cardiac-output-guided strategy without an explicit CI target (OPTIMISE II Trial Group 2024). Implementation across different surgical settings may additionally be limited by differences in available monitoring technologies, staff experience, protocol adherence, and the potential adverse effects associated with fluids, vasopressors, and inotropic drugs. Taken together, the current evidence supports physiological and clinical plausibility but does not establish an indication for widespread CI-targeted GDT. Further research in the field should focus on using homogeneous definitions of postoperative complications to empower comparability of studies. Especially the wide prediction interval suggests potential and relevant heterogeneity may uncover confounding factors, such as specific risk constellations leading to some patients profiting more than others or experiencing harm from the CI-GDT respectively. Furthermore, the cut-off for inotropic support should be investigated. On a physiologic basis it seems reasonable to support cardiac function if relevant cardiac impairment is detected perioperatively. However, we should carefully assess the border of real cardiac impairment and physiologic adaptation to decrease perioperative metabolic demand. As Dobutamine is the major difference between the therapy regimes (standard care vs. intervention) the impact of the drug per se outside of cardiac surgery should also be investigated. In certain scenarios patients did not profit from CI-GDT, therefore we should also consider the side effects of Dobutamine.

This review has several limitations. The included evidence was derived from multiple relatively small trials involving clinically heterogeneous patient populations undergoing a wide range of surgical procedures. The haemodynamic interventions also varied substantially with respect to the predefined CI thresholds, additional target variables, monitoring technologies, and protocol-specified use of fluids, vasopressors, and inotropic drugs. Furthermore, definitions and observation periods for postoperative complications and mortality were not uniform across studies. These sources of clinical and methodological heterogeneity probably contributed to the observed statistical heterogeneity and the wide prediction interval. Consequently, the pooled estimate should be interpreted as an average association across heterogeneous multimodal treatment algorithms, and its generalisability to individual surgical populations and clinical settings remains limited. Because CI was not assessed as an isolated intervention, the incremental effect attributable specifically to CI targeting cannot be determined.

The questions arising from this study are.

- What perioperative value is reasonable for the initiation of inotropic support to improve postoperative outcome?
- Which patients can profit the most from CI-GDT and which patients might experience harm?
- Is there a difference depending on the type of operation considering postoperative outcome and CI-GDT?

Rather than simply requiring another large trial of CI-GDT versus usual care, future research should determine whether incorporating CI into contemporary haemodynamic algorithms provides incremental clinical benefit, and whether specific high-risk patient populations or haemodynamic phenotypes can be identified in whom such targeting is advantageous.

## Conclusion

GDT algorithms incorporating an explicit CI target were associated with a lower incidence of postoperative complications compared with usual care, whereas mortality was not significantly different. However, substantial heterogeneity and the prediction interval crossing the null limit the generalisability of the pooled benefit. These findings do not establish an independent benefit of CI targeting over other GDT strategies. Future research should clarify whether incorporation of CI provides incremental benefit within contemporary haemodynamic algorithms and identify patient populations most likely to benefit.

## Supplementary Information

Supplementary Material 1.

Supplementary Material 2: Supplementary Figure S1. Leave-one-out sensitivity analysis for the primary outcome of participants experiencing at least one postoperative complication. Each point represents the pooled log odds ratio after omission of the indicated study. The red dashed line represents the overall pooled effect estimate based on all 20 studies. Exclusion of any individual study did not materially alter the pooled effect estimate, indicating that the overall result was not driven by a single study. OR, odds ratio.

Supplementary Material 3: Supplementary Figure S2. Funnel plot for the primary outcome of participants experiencing at least one postoperative complication. Study-specific effect estimates are plotted as log odds ratios against their standard errors. The vertical dotted line represents the pooled effect estimate, and the diagonal boundaries indicate the approximate 95% confidence limits. Egger’s test showed no evidence of funnel-plot asymmetry (z = − 1.44, p = 0.15), and the trim-and-fill analysis did not impute any additional studies. OR, odds ratio.

## Data Availability

All data analyzed during this study are included in the published articles and their supplementary information files. Extracted data and statistical code used for the meta-analysis are available from the corresponding author on reasonable request.
