# Supplementary material for: Effect of cardiac-index guided intraoperative haemodynamic therapy on postoperative outcome in non-cardiac surgery patients – a systematic review and meta-analysis
Source: Perioper Med (Lond). 2026 Nov 7;15:96. doi: 10.1186/s13741-026-00760-8 (PMC13744102; doi:10.1186/s13741-026-00760-8)
Supplement: Supplementary file 1 [file 13741_2026_760_MOESM1_ESM.docx]

Supplement material

Search strategy:

Population:

“Perioperative Care”[MeSH], “Monitoring, Intraoperative”[MeSH], “Surgical Procedures, Operative”[MeSH]

Intervention (Cardiac Index-guided therapy):

“Haemodynamic Monitoring”[MeSH], “Cardiac Output”[MeSH], “Cardiac Output, Low”[MeSH], “Cardiac Function”[MeSH], “Therapeutics”[MeSH]

Comparator (Conventional monitoring):

“Monitoring, Physiologic”[MeSH], “Standard of Care”[MeSH], “Usual Care”, “Routine Monitoring”, “Standard Monitoring”

Outcomes:

“Treatment Outcome”[MeSH], “Patient Outcome Assessment”[MeSH]

Study type filters:

(“Meta-Analysis”[Publication Type] OR “Review”[Publication Type])

Additional filters may include: “Comparative Study”[Publication Type], “Randomised Controlled Trial”[Publication Type] if appropriate.
